# Biomarkers of systemic inflammation and depression and fatigue in moderate clinically stable COPD

**DOI:** 10.1186/1465-9921-12-3

**Published:** 2011-01-05

**Authors:** Khaled Al-shair, Umme Kolsum, Rachel Dockry, Julie Morris, Dave Singh, Jørgen Vestbo

**Affiliations:** 1 University Of Manchester, Medicines Evaluation Unit, NIHR Translational Research Facility, Manchester Academic Health Sciences Centre, University Hospital Of South Manchester Foundation Trust, Wythenshawe, Manchester, UK; 2 The Medical Statistics Department, Education and Research Centre, South Manchester University Hospital, Wythenshawe, The University of Manchester, UK; 3 Department of Cardiology and Respiratory Medicine, Hvidovre University Hospital, Hvidovre, Denmark

## Abstract

**Introduction:**

COPD is an inflammatory disease with major co-morbidities. It has recently been suggested that depression may be the result of systemic inflammation. We aimed to explore the association between systemic inflammation and symptoms of depression and fatigue in patients with mainly moderate and clinically stable COPD using a range of inflammatory biomarkers, 2 depression and 2 fatigue scales.

**Method:**

We assessed 120 patients with moderate COPD (FEV_1_% 52, men 62%, age 66). Depression was assessed using the BASDEC and CES-D scales. Fatigue was assessed using the Manchester COPD-fatigue scale (MCFS) and the Borg scale before and after 6MWT. We measured systemic TNF-α, CRP, TNF-α-R1, TNF-α-R2 and IL-6.

**Results:**

A multivariate linear model of all biomarkers showed that TNF-α only had a positive correlation with BASDEC depression score (p = 0.007). TNF-α remained positively correlated with depression (p = 0.024) after further adjusting for TNF-α-R1, TNF-α-R2, 6MWD, FEV_1_%, and pack-years. Even after adding the MCFS score, body mass and body composition to the model TNF-α was still associated with the BASDEC score (p = 0.044). Furthermore, patients with higher TNF-α level (> 3 pg/ml, n = 7) had higher mean CES-D depression score than the rest of the sample (p = 0.03). Borg fatigue score at baseline were weakly correlated with TNF-α and CRP, and with TNF-α only after 6MWT. Patients with higher TNF-α had more fatigue after 6MWD (p = 0.054).

**Conclusion:**

This study indicates a possible association between TNF-α and two frequent and major co-morbidities in COPD; i.e., depression and fatigue.

## Introduction

COPD is a chronic inflammatory disease with systemic manifestations such as muscle wasting, depression and fatigue [1]. Systemic manifestations of COPD may significantly affect patients' quality of life and the prognosis of the disease [1]. It has been suggested that systemic manifestations may be related to systemic inflammation in COPD [2,3].

Depression is a major comorbidity in COPD; it is associated with poor functional performance [4], significant impairment in health status and high mortality [5]. Fatigue is one of the most prominent disabling symptoms in COPD [6]. It is strongly associated with depression [7], decline in daily functional activity [6], and substantial impairment in quality of life [8].

The association between symptoms of depression and fatigue and systemic inflammation has been examined in depth in healthy individuals and in illnesses such as coronary heart disease (CHD) [9-11]. In COPD, patients with more systemic inflammation as well as more depression or fatigue have been shown to be less physically active and more exercise intolerant [4,6,12]. Recently, Barnes and Celli have speculated that depression may correlate with systemic inflammation [13] and to date no study has addressed this.

We aimed to explore the association between systemic inflammation and symptoms of depression and fatigue in patients with mainly moderate and clinically stable COPD using a range of inflammatory biomarkers, two depression scales and two fatigue scales.

## Methods

### Study subjects

The subjects enrolled in this study were 120 clinically stable patients enrolled from outpatient clinics and advertisements. More information on the recruitment, inclusion and exclusion criteria, and patients' demographic data has been described previously [4]. Briefly, all patients had COPD according to GOLD [14] and had been clinically stable for at least 4 weeks. Patients with exacerbations in the last 4 weeks were either rescheduled or excluded. We excluded patients with current or recurrent symptomatic ischemic heart disease, congestive heart disease, cerebrovascular disease, dementia, lung cancer, known psychiatric illness, maintenance treatment with systemic corticosteroids (oral, parenteral), active tuberculosis, inflammatory bowel syndrome or insulin-dependent diabetes mellitus. All participants gave written informed consent to participate in the study, and the South Manchester Research Ethics Committee had approved the study (Reference number 05/Q1402/41).

### Assessments

For assessment of depression, two instruments were used: The Brief Assessment Schedule Depression Cards (BASDEC) and the Centre for Epidemiological Study on Depression (CES-D) Scale [15,16]. Both of the scales have been frequently used in assessing depression in COPD [17-19]. The impact of fatigue was assessed using our Manchester COPD fatigue scale (MCFS) which has a high level of validity and reliability [7]. It measures total fatigue as well as dimensional assessment of physical, cognitive and psychosocial fatigue. The total score ranges from 0-54, the higher the score the more the fatigue. We also assessed the intensity of fatigue before and after a 6 minute walk test (6MWT) using the Borg scale [20]. Patients rated their feeling by the selection of one option, ranging from 0-12, with 0 meaning no fatigue and 12 meaning extreme fatigue intensity. We used the Bioelectrical Impedance Analysis (BIA) to measure body composition by (Bodystat Ltd, Douglas, UK). Spirometry was done according to the ATS/ERS Standardisation Guideline [21] using a Jaeger MasterScreen spirometer (Jaeger Ltd, Hoechberg, Germany). Functional performance was measured using the 6MWT according to the ATS guideline [22]. Health Status was measured by the St George's Respiratory Questionnaire (SGRQ) [23].

### Systemic biomarkers measurement

Venous blood samples were obtained before the exercise test to measure the required biomarkers. The samples were centrifuged and allocated in well-marked tubes with patients' initials, date of donation, database number and type of sample (plasma or serum), and samples were immediately stored at -80°C until analysis. Plasma TNF-α and serum IL-6 was measured by high sensitivity ELISA (Quantikine, R&D Systems Europe, Oxon, UK) with a lower limit of detection of 0.5 pg/ml and 0.156 pg/ml respectively. Plasma CRP was measured by high sensitivity particle-enhanced immunonephelometry (Cardiophase; BN systems, Dade Behring, Newark, NJ, USA).

### Statistical analysis

Normal distribution was assessed by Kolmogorov-Smirnov goodness of fit test and non-parametric data were natural log transformed or presented as median and interquartile range (IQR). The univariate correlation of biomarkers and depression and fatigue scores were examined by Spearman correlation. The difference in the mean of parametric variables was examined using the analysis of variance (ANOVA). The Mann-Whitney and Kruskal-Wallis tests were used to examine the difference in the median value of each biomarker in two groups or quartiles of either depression or fatigue respectively. The chi square (*x*^2^) test was used to examine the categorical association of systemic biomarkers and depression and fatigue. The multivariate linear and binary regression analyses were used to examine the association of factors with depression or fatigue. SPSS version 15 (SPSS Inc, USA) was used.

## Results

We examined 120 patients with mainly moderate COPD (mean FEV_1_% 52.5 (SD 18.5)), mean age was 66 years and women made up 38% of the sample. Patients with GOLD stage 2 (60 (50%)) dominated the sample while patients with GOLD stage 1, 3 and 4 represented (6 (5%), 38 (32%) and 16 (13%)), respectively. Although the majority of the patients were ex-smokers (86 (71.7%), there were 34 current smokers (28.3%). The current smokers were slightly younger, half of them were women, and they had slightly worse airway obstruction, more fatigue, more depressive symptoms and less lean tissue. More demographic data are shown in table 1. The median (IQR) of BASDEC and CES-D scores were 3 (4.5) and 10 (12) respectively, and the mean (SD) MCFS was 24.8 (12.8) and the median (IQR) Borg scale at baseline and post-6MWT were 0.5 (2) and 2 (3.5) respectively.

**Table 1:** Baseline characteristics of the sample; mean values and standard deviations are shown unless otherwise noted

	All	Ex-smokers	Current smokers	P - value
Number	120	86 (71.7%)	34 (28.3%)	

Age, yrs	66 ± 6.7	67 ± 6.9	64 ± 6.6	0.03

Females (%)	46 (38%)	30 (35%)	16 (47%)	0.4*

FEV_1 _%	52.5 ± 18.5	53.5 ± 18.5	47.8 ± 17.7	0.12

PaO_2 _(kPa)	9.2 ± 1.4	9.1 ± 1.1	9.3 ± 1.2	0.4

PaCO_2 _(kPa)	5.2 ±.58	5.1 ± 0.5	5.3 ± 0.7	0.05

MCFS	25.1 ±12.5	23.9 ± 12	28 ± 13.5	0.11

CES-D Median (IQR)	10 (12)	9 (12)	11 (11)	0.4#

BMI (kg/m^2^)	27.5 ± 5.8	27.9 ± 5.5	26.5 ± 6.4	0.22

FFMI (kg/m^2^)	17.8 ± 3.1	18.3 ± 3.2	17.2 ± 3.6	0.09

Pack/years Median (IQR)	40 (25.8)	38.3 (31.1)	41.9 (22.5)	0.8#

# Mann-Whitney U Test; * *x*^2^-Test; IQR = Interquartile Range; FEV_1 _% = Forced Expiratory Volume over 1 second of predicted; PaO_2 _= Arterial oxygen partial pressure; kPa = kilo Pascal; PaCO_2 _= Arterial carbon dioxide pressure; MCFS = Manchester COPD Fatigue Scale; CES-D = Centre for Epidemiologic Studies Depression Scale; BMI = Body Mass Index; FFMI = Fat-Free Mass Index.

There were mild to moderate intercorrelations between the systemic inflammatory biomarkers as shown in table 2.

**Table 2:** Univariate (Spearman (rho)) correlations between inflammatory biomarkers

	TNF-α	CRP	TNF R1	TNF R2
TNF-**α**	1			

CRP	0.11	1		

TNF-**α **R1	0.368**	0.285**	1	

TNF-**α **R2	0.282**	0.104	.548**	1

IL-6	0.114	0.449**	.391**	.199*

** Correlation is significant at the 0.01 level (2-tailed).

* Correlation is significant at the 0.05 level (2-tailed).

**CRP = **C-reactive protein; IL-6 = Interleukin-6; **TNF-α = **Tumor necrosis factor-α; **TNF-α R1 = **Tumor necrosis factor-α receptor1; **TNF-α R2 = **Tumor necrosis factor-α receptor2

### Depression and systemic inflammation

Univariate correlation analyses showed no statistically significant association between systemic inflammatory biomarkers and CES-D and BASDEC depression scores except for a weak correlation between TNF-α- R1 and BASDEC score (rho = -0.2, p = 0.03). We found no difference in the median of all biomarkers between symptomatically depressed and not depressed.

Using the BASDEC scores as the dependent variable, a multivariate linear regression analysis showed a positive association between TNF-α and depression scores (Beta = 0.26, p = 0.007) as shown in table 3 (Module 1). The association remained unchanged after adjusting for TNF-α-R1 and TNF-α-R2 (Beta = 0.27, p = 0.005). TNF-α still had a positive correlation with depression (Beta = 0.23, p = 0.024) in the multivariate model after further adjusting for 6MWD, FEV_1_%, and pack/years, as shown in table 3 (Module 2); this model explained 15.6% of the variance in depression scores where TNF-α alone contributed with 5%. Furthermore, adding the total Manchester COPD Fatigue Scale (MCFS) score, BMI and FFMI to the model did not change the findings; i.e., TNF-α still had a significant positive correlation with BASDEC depression score (Beta = 0.17 p = 0.044).

**Table 3:** Multivariate linear regression modules for factors associated with depression

Module 1			Module 2		
**Variables**	**Beta**	**p**	**Variables**	**Beta**	**p**

TNF-α	0.26	0.007	TNF-α	0.23	0.024

CRP	0.07	0.5	TNF-α-R1	-0.13	0.3

TNF-α-R1	-0.1	0.5	TNF-α-R2	-0.13	0.3

TNF-α-R2	-0.2	0.2	6MWD	-0.22	0.027

IL-6	-0.2	0.1	FEV_1_%	0.02	0.9

			Pack/years	-0.02	0.9

*BASDEC depression score was the dependent variable

**CRP = **C-reactive protein; **FEV_1 _= **Forced Expiratory Volume over 1 Second; IL-6 = Interleukin-6; **6MWD = **6 Minute Walk Distance; **TNF-α = **Tumor necrosis factor-α; **TNF-α R1 = **Tumor necrosis factor-α receptor1; **TNF-α R2 = **Tumor necrosis factor-α receptor2.

**Figure 1:**
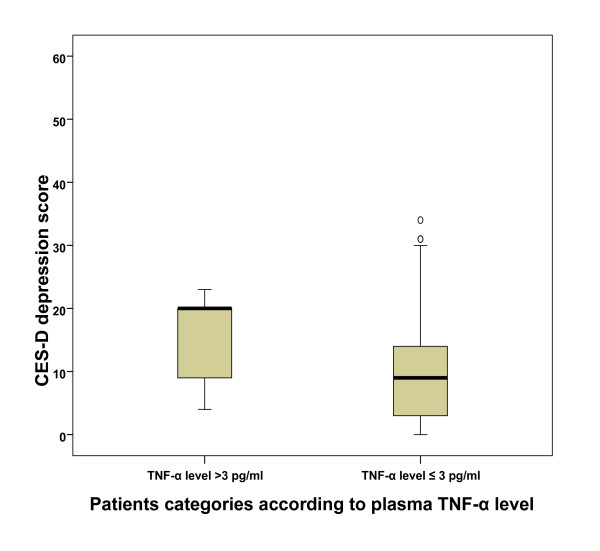
**Mean CES-D depression scores in relation to plasma TNF-α level**. **CES-D **= Centre for Epidemiologic Studies Depression Scale, **TNF-α = **Tumor necrosis factor-α

For exploration, we selected higher cut-off points for each systemic biomarker to categorize the sample, and we found that the patients with higher TNF-α level (> 3 pg/ml, n = 7) had higher mean CES-D depression score than the rest of the sample (15 vs 10.4, p = 0.03) as shown in figure 1. For BASDEC scores the differences were less obvious (5.4 vs 3.6, p = 0.2). We found no statistically significant differences for CRP and IL-6.

### Fatigue and systemic inflammation

We found no statistically significant correlation between total MCFS scores and any of the systemic biomarkers. Similarly, no univariate statistically significant correlation was found between systemic biomarkers and the physical, cognitive or psychosocial dimensions of MCFS (p > 0.05 for all correlations). Using categorical analyses, there was no statistically significant difference in the median of all biomarkers between the 4 fatigue quartiles. Similarly, we found no statistically significant difference in the median of the inflammatory biomarkers in quartiles of physical and cognitive.

Borg fatigue scores at baseline had a weak positive correlation with TNF-α and CRP (rho = 0.24, p = 0.01, and 0.19, p = 0.05, respectively). Similarly, Borg fatigue scores after 6MWT had a weak correlation with TNF-α only (rho = 0.23, p = 0.01).

For further exploration, we selected higher cut-off points and found that the mean of the total and dimensional MCFS score of patients with higher TNF-α (> 3 pg/ml, n = 7) was higher than the rest of the sample, but the differences did not reach statistical significance. A similar trend was seen for the mean fatigue score in Borg scale after 6MWT (3.7 vs. 2.2, p = 0.054). However, this correlation was not found in multivariate analyses.

## Discussion

We explored the association between systemic inflammation and depression and fatigue using a range of inflammatory biomarkers, two depression scales and two fatigue scales in a cohort of 120 patients with clinically stable COPD. There were modest correlations between systemic inflammation and depression and fatigue. However, the association between TNF-α and depression remained significant even after adjusting for confounding factors in multivariate analyses. This finding was also consistently seen in further categorical analyses.

An association between systemic inflammation and depression could be the result of the effect of confounders. It has been suggested that both systemic inflammation and depression correlate with poor functional performance [4,24], fatigue [10,25], BMI [24,26] and FFMI [4,27]. To explore the possibility of confounding as a result of these factors, we did different multivariate analyses. In a model with depression as the dependent variable, adjusting for the MCFS score, 6MWD, FEV_1_%, BMI and FFMI did not markedly change the association between TNF-α and depression and it remained statistically significant. The robustness of this association may reflect a true association as a result of the fact that COPD is principally a progressive inflammatory disabling disease [1] and that major depression or sub-threshold depressive symptoms are quite prevalent [17,28] even in patients with moderate clinically stable COPD [4]. Therefore, it seems plausible that systemic inflammation is correlated with depression as suggested by Barns and Celli [13].

The mechanism behind this relationship could have a bidirectional nature [29] particularly in COPD. In fact, studies have shown that inflammatory cytokines had a direct effect on the central nervous system including the enhancement of negative moods [29-32]. On the other hand, depression was associated with increased plasma cytokines, and the production of pro-inflammatory cytokines was frequently seen in depression [29,30]. This could be clinically important given the chronic inflammatory progressive pattern of COPD, and opens the possibility of effective antidepressants having an effect on the inflammatory response system [31,33] or effective anti-inflammatory therapy having an effect on depression, a major comorbidity in COPD.

We used our validated Manchester COPD-fatigue scale (MCFS) [7] to assess the association between systemic inflammation and fatigue and we found that patients with less fatigue had a tendency to have lower levels of TNF-α. We found this for both the total and dimensional fatigue using the MCFS. Moreover, there was a weak association between the severity (intensity) of fatigue before and after exercise test with TNF-α and CRP but not with TNF-α-Rs and IL-6. It has previously been reported that exhausted patients with CHD had higher levels of TNF-α and IL-6 (mean rank of TNF-α and IL-6 values of exhausted vs not exhausted were 17.9 vs 12.3, p = 0.04, and 17 vs 12, p = 0.06, respectively) [10].

Even with the significant correlations reported, the small association between systemic inflammation and these systemic manifestations should be discussed. First, the sample of this study composed of mainly moderate COPD and a larger sample with a range of COPD severities would better explore this possible relationship. Secondly, we have chosen stable patients and the signal may be more apparent in frequent exacerbators or patients with major depression who are unlikely to be in this study population. For instance, a worse scenario would be expected had we looked at patients with less stable patients or even patients during exacerbation, given that others have found exacerbations are correlated with systemic inflammation [34], depression [19] and fatigue [25]. More studies, probably of longitudinal nature will be required to disentangle these associations.

We can not preclude that our findings may be affected by the variability in the measured biomarkers [10,35,36]. However, we measured a range of biomarkers that have been shown to be important in studying the morbidity and mortality in COPD [37], and we used several well-validated subjective and objective assessment tools. We applied strict inclusion criteria by excluding patients with diseases that may have a potential confounding effect. We made the effort to ensure that the blood samples were obtained carefully from clinically stable patients. For this purpose, we excluded patients with recent symptomatic coronary heart disease and patients with COPD exacerbations were either rescheduled or excluded.

## Conclusion

In conclusion, our data indicate an association between TNF-α and two major co-morbidities in COPD; i.e., depression and fatigue. However, further studies are required to explore this subject and to tackle the biological roles of these biomarkers in relation to depression and/or fatigue.

## Abbreviations

**(BMI)**: Body Mass Index; **(BODE)**: Multidimensional index (B = Body mass index, O = Obstruction of air ways as measured by FEV_1_, D = Dyspnoea as measured by MRC scale, E = Exercise capacity as measured by 6MWT); **(BASDEC)**: Brief Assessment Schedule Depression Cards; **(CES-D)**: Centre for Epidemiologic Studies Depression Scale; **(COPD)**: Chronic obstructive pulmonary disease; **(CRP)**: C-reactive protein; **(FFMI)**: Fat-Free Mass Index; **(FEV_1_)**: Forced Expiratory Volume in 1 Second; **(FVC)**: Forced Vital Capacity; **(GOLD)**: Global Initiative for Chronic Obstructive Lung Disease; **(IL-6)**: Interleukin-6; **(IQR)**: Interquartile range; **(L)**: Litre; **(MRC)**: Medical Research Council Scale; **(6MWD)**: 6 Minute Walk Distance; **(6MWT)**: 6 Minute Walk Test; **(m)**: Meter; **(Q)**: Quartile; **(TNF-α)**: Tumor necrosis factor-α; **TNF-α R1**: Tumor necrosis factor-α receptor1; **TNF-α R2**: Tumor necrosis factor-α receptor2.

## Competing interests

The authors declare that they have no competing interests.

## Authors' contributions

KA participated in the study design and data collection and performed all the statistical analyses and wrote the manuscript. UK participated in data collection and analysis. RD participated in data collection and analysis. JM participated in data analysis and manuscript review. DS participated in the study design, data analysis and manuscript review. JV participated in the study design, data analysis and manuscript writing and review. All authors read and approved the final manuscript.
